# HIV/AIDS and health services: misaligned policy and maligned subjects

**DOI:** 10.1186/1753-6561-6-S5-O16

**Published:** 2012-09-28

**Authors:** Aalya Amin, Javaid Rashid

**Affiliations:** 1 Jamia Millia Islamia University, Delhi, India; 2 Jawaharlal Nehru University, Delhi, India

## Introduction

HIV/AIDS has often been described as the most contemporary global health concern. The understanding of HIV/AIDS has come a long way from it being largely identified as a disease of certain selected groups (based on their region, ethnicity, behaviour and sexual orientation) and Africa as the epicentre of the epidemic to it being a transnational disease which has its roots in colonialism, poverty, gender discrimination and racism. AIDS as a global concern and as a significant international-relations issue has mobilized institutions and resources all over the world. Many countries have made huge allocations for AIDS prevention programmes and many policies and programmes have been formulated to reduce the rates of HIV transmission. But the way HIV/AIDS has been conceptualized in these policies, reflects a very positivist, de-contextualized and narrow biomedical approach. A lot of focus has been on ‘behavioural therapy/change programmes’ and promotion of safe sex. It is anticipated that awareness of HIV and promotion of condom use will help people practice safe behaviour and thus reduce the transmission. Such an understanding fails to consider the fact that behaviours are patterned by social, cultural and economic circumstances, which cannot be changed or altered by information, which is de-contextualized and insensitive to varied social settings.

The paper examines the social implications of HIV/AIDS and how it impacts the infected individuals, their health, interpersonal, societal relations and their livelihood. By analysing the accounts of people living with HIV/AIDS, the paper seeks to capture their lived experiences. It also attempts to analyse the relationship of HIV/AIDS prevention as a Millennium Development Goal with other Millennium Development Goals.

## Methods

The study was conducted in Delhi. As it was quite difficult to locate and approach the HIV infected-people, the organizations working with people living with HIV/AIDS were approached. This facilitated the interaction and meetings with the respondents.

Twenty cases were selected considering the efficiency and requirements of the research. Recruitment into the study was on a voluntary basis with the oral consent of the individual. Respondents were assured of anonymity and were encouraged to terminate the discussions if and when they deemed it appropriate to do so. To illuminate the ways people make sense of, and live with HIV/AIDS, this study adopted an interpretative phenomenological approach in analyzing the data.

## Results and discussion

It was found that targeted and a compartmentalized approach to HIV prevention has led to stigmatization and further marginalization of people living with HIV/AIDS. The suffering due to HIV/AIDS is significantly linked to poverty, inequality and other socio-cultural determinants. It is argued that there needs to be a comprehensive programme that conceptualizes, contextualizes and integrates HIV prevention with other millennium development goals like poverty alleviation and improving reproductive health. Through an exclusive focus only on reducing the infection rates, international and national policies fail to deliver care, support and importantly *‘*livelihood’ for the people infected. It is the multi-faceted suffering of infected people that goes unnoticed and hence neglected in current efforts to prevent HIV/AIDS.

## Competing interests

Authors declare that they have no conflict of interest.

## Funding statement

The authors funded the study.

